# Boosting facility deliveries with results-based financing: a mixed-methods evaluation of the government midwifery incentive scheme in Cambodia

**DOI:** 10.1186/s12884-015-0589-x

**Published:** 2015-08-15

**Authors:** Por Ir, Catherine Korachais, Kannarath Chheng, Dirk Horemans, Wim Van Damme, Bruno Meessen

**Affiliations:** National Institute of Public Health, Ministry of Health, PO BOX 1300, Phnom Penh, Cambodia; Department of Public Health, Institute of Tropical Medicine, Antwerp, Nationalestraat 155, B-2000 Antwerp, Belgium; Health Sector Support Programme, Belgian Technical Cooperation, Phnom Penh, Cambodia

## Abstract

**Background:**

Increasing the coverage of skilled attendance at births in a health facility (facility delivery) is crucial for saving the lives of mothers and achieving Millennium Development Goal five. Cambodia has significantly increased the coverage of facility deliveries and reduced the maternal mortality ratio in the last decade. The introduction of a nationwide government implemented and funded results-based financing initiative, known as the Government Midwifery Incentive Scheme (GMIS), is considered one of the most important contributors to this. We evaluated GMIS to explore its effects on facility deliveries and the health system.

**Methods:**

We used a mixed-methods design. An interrupted time series model was applied, using routine longitudinal data on reported deliveries between 2006 and 2011 that were extracted from the health information system. In addition, we interviewed 56 key informants and performed 12 focus group discussions with 124 women who had given birth (once or more) since 2006. Findings from the quantitative data were carefully interpreted and triangulated with those from qualitative data.

**Results:**

We found that facility deliveries have tripled from 19 % of the estimated number of births in 2006 to 57 % in 2011 and this increase was more substantial at health centres as compared to hospitals. Segmented linear regressions showed that the introduction of GMIS in October 2007 made the increase in facility deliveries and deliveries with skilled attendants significantly jump by 18 and 10 % respectively. Results from qualitative data also suggest that the introduction of GMIS together with other interventions that aimed to improve access to essential maternal health services led to considerable improvements in public health facilities and a steep increase in facility deliveries. Home deliveries attended by traditional birth attendants decreased concomitantly. We also outline several operational issues and limitations of GMIS.

**Conclusions:**

The available evidence strongly suggests that GMIS is an effective mechanism to complement other interventions to improve health system performance and boost facility deliveries as well as skilled birth attendance; thereby contributing to the reduction of maternal mortality. Our findings provide useful lessons for Cambodia to further improve GMIS and for other low-income countries to implement similar results-based financing mechanisms.

## Background

Skilled attendance at birth is considered one of the most critical interventions for ensuring safe motherhood [1]. In addition to skilled attendance, it is important that mothers give birth in an appropriate setting, e.g. a health facility, where hygienic conditions, supplies and equipment can help reduce risk of complications. Adequate transport and effective communication systems for timely referral for emergency obstetric care when complications arise is also extremely important [2, 3]. However, in many low-income countries, despite considerable national and global efforts to improve safe motherhood services, the coverage of facility deliveries remains low and the rates of decline in maternal mortality ratio, if any, are insufficient to achieve Millennium Development Goal five (MDG 5) [4].

Cambodia has significantly increased the coverage of facility deliveries and reduced the maternal mortality ratio in a relatively short-time span. The Cambodia Demographic and Health Surveys (CDHS) [5, 6] showed that the coverage of facility deliveries rose from 22 % in 2005 to 54 % in 2010, whereas the maternal mortality ratio significantly decreased from 473 (95 % CI: 338–605) to 206 (95 % CI: 124–288) maternal deaths per 100,000 live births in the same period. This achievement resulted from concerted efforts in strengthening the public health system to supply essential reproductive and maternal health services and to remove barriers to accessing these services for pregnant women. The introduction of the Government Midwifery Incentive Scheme (GMIS) was one of these efforts [7, 8]. Implemented nationwide and funded by the government, GMIS aimed to boost facility deliveries by paying midwives and other trained health personnel with cash incentives based on the number of live births they attended in public health facilities—USD15 for a live birth in a health centre and USD10 for a live birth in a referral hospital. The reason for the higher payment in a health centre compared to a hospital was to provide a stronger incentive for deliveries at health centres—the largest primary health care network and the recommended place for normal deliveries.

It is generally believed that linking payments with results or performance targets, commonly known as results-based financing (RBF), can stimulate health providers and users to achieve the results or targets. This in turn contributes to improving health system performance and health outcomes. RBF is defined as “*a cash payment or non-monetary transfer made to a national or sub-national government, manager, provider, payer or consumer of services after predefined results have been attained and verified*”. RBF includes a wide range of approaches or groups of interventions that vary greatly according to the objectives, the targeted behaviours (or indicators), the entity receiving the reward and the type and magnitude of the financial reward [9]. Some RBF approaches in health focus on improving the provision of health services (supply-side RBF) or on increasing the uptake of health services (demand-side RBF), whereas others address both supply and demand barriers (a supply-side and demand-side mixed RBF). GMIS is a form of supply-side RBF through which the government links its budget funds to desired outputs, rather than just financing inputs, which is commonly known as results-based budgeting [10, 11]. Such supply-side RBF is often implemented in combination with demand-side RBF, such as conditional cash transfers [12–14].

Despite increasing evidence showing the positive effects of RBF on health services utilization or coverage, whether such strategy really helps improve health system performance and health outcomes, especially maternal and child health, is still a greatly debated subject [10, 15–17]. A part from its apparent positive effects, anecdotal evidence shows that there are also limitations and pitfalls related to the implementation of such schemes.

With a mixed-methods design, we conducted the first evaluation of GMIS, with the aim of exploring the effects of this scheme on deliveries attended by trained health professionals in public health facilities (facility deliveries) and possible spill-over effects on the whole health system, mainly at the district level, and vice versa. It has been argued that such system effects are important attributes of RBF [16]. We also identified the limitations of GMIS and possible challenges ahead. This assessment help to fill the evidence gap and generate lessons for Cambodia and other low-income countries contemplating to implementation of similar RBF mechanisms to achieve MDG5.

## Methods

### Study setting

Cambodia is a low-income country in the South-East Asian region with a population of 14.3 million inhabitants, of which 80 % live in rural areas, largely relying on agriculture. According to the 2007 Cambodia Socio-Economic Survey, 30 % of the population live below the national poverty line of USD0.59 per day [18]. Over the past decade, the country has made substantial progress in improving the health of the population, as evidenced by the changes in key health indicators, especially reproductive, maternal and child health indicators, reported in the Cambodian Demographic and Health Surveys (CDHS) 2000, 2005, and 2010 (Table 1). However, Cambodia’s health indicators remain relatively low if compared with other countries in the region and are inequitably distributed among different socio-economic groups.

**Table 1:** Maternal and child health related indicators

*Indicators*	*CDHS 2000*	*CDHS 2005*	*CDHS 2010*
Children 12–23 months fully vaccinated (%)	40	67	79
Use of modern contraceptive method (%)	19	27	35
Antenatal care at least once by trained personnel (%)	38	69	89
Deliveries in health facilities (%)	10	22	54
Deliveries assisted by trained personnel (%)	32	44	71
Exclusive breastfeeding (%)	11	60	74
Total fertility rate	4.0	3.4	3.0
Infant mortality per 1,000 live births	95	66	45
Under 5 mortality per 1,000 live births	124	83	54
Maternal mortality ratio per 100,000 live births	437	472	206

Source: CDHS Reports 2000, 2005 and 2010

*CDHS* means Cambodia Demographic and Health Survey

The Cambodian public health care system is composed of operational health districts. Each health district has a number of health centres and a referral hospital, respectively providing first and second line health services to a population of 100,000-200,000. By 2011, there were 1,024 health centres providing primary health care and 79 referral hospitals in 77 health districts, providing a reasonable physical coverage throughout the country [19]. Next to this public sector, there is a thriving private sector which firmly occupies the most lucrative segments of the health care market [20].

Since 1996, several reform initiatives have been undertaken to improve access for the population to priority public health services, especially maternal services. In addition to human resource development and reforms, Cambodia has been particularly creative in introducing innovative health financing schemes. The major ones include ‘contracting’ and other performance-based financing schemes [21–23], health equity funds [24–30], vouchers [8, 31], community-based health insurance, and the Government Midwifery Incentive Scheme (GMIS). Available evidence shows that these major health financing schemes, in particular vouchers [31] have also contributed to the increase in facility deliveries.

### Study intervention—the Government Midwifery Incentive Scheme

The Government Midwifery Incentive Scheme (GMIS) is the most recent health financing scheme which specifically addresses maternal health service challenges. It is a supply-side results-based health financing mechanism aimed at motivating skilled birth attendants to promote deliveries in public health facilities (facility deliveries), thereby contributing to the reduction of maternal mortality. The most remarkable feature of GMIS lies in the ‘G’: the scheme is fully implemented by the Royal Government of Cambodia with its own funds, straight nationwide. Low remuneration of midwives which was increasingly recognised as the main cause of low facility deliveries in Cambodia triggered discussions among policy makers to look for solution. The discussions resulted in an agreement to upgrade the government salary scale for midwives and provide them with cash incentives. This was publicly announced by the Prime Minister in early 2007. Subsequently, the Ministry of Economy and Finance and the Ministry of Health jointly issued a *Prakas* (directive) on 02 April 2007 to allocate government budget to provide incentives for midwives at an amount of USD15 for each live birth attended in health centres and USD10 in hospitals [32]. On 28 June 2007 the Ministry of Health issued a circular providing guidance on the implementation and monitoring of GMIS [33]. The circular stipulated that besides midwives, physicians and other trained health personnel can also receive these incentives when attending deliveries in public health facilities. Up to 30 % of the incentives have to be shared with other health personnel in the facility and eventually with other people such as traditional birth attendants who refer women to the facility for delivery. The number of deliveries is reported monthly by health facilities through the routine health information system. The report must be signed by the director of the health facility and, for health centres, also by the commune chief. Based on the number of reported deliveries, incentives are disbursed quarterly to the facilities through public financial disbursement channels. GMIS became operational nationwide in October 2007, when midwives and other health personnel began to receive the incentives.

### Study design

This is a retrospective impact evaluation that was conducted in early 2012, more than four years after the start of GMIS. In this study, we investigated: (i) whether and to what extent GMIS contributes to increased facility deliveries and/or deliveries attended by trained health personnel; (ii) whether GMIS contributed to improving the district health system in terms of infrastructure, availability and commitment of midwives and other personnel, health service organisation at the facilities, referrals between villages, health centres and referral hospitals, and health centre supervisions by the health district; (iii) in which district health system context (e.g. districts with and without other major health financing interventions) GMIS was most effective; and (iv) the limitations and pitfalls of GMIS, including its unintended effects on the district health system.

These questions are different in nature and answers to such questions thus require varied approaches to data collection and analysis. Therefore, we used a mixed-methods design, which allows collecting, analysing, and interpreting quantitative and qualitative data in a single study or in a series of studies that investigate the same underlying phenomenon [34, 35]. Such approach is increasingly used for impact evaluations [36]. In the absence of control data, we adopted an interrupted time series design, one of the most robust quantitative methods for impact evaluation [37], to analyse routine longitudinal data extracted from the national health information system with the aim of assessing the impact of GMIS on facility deliveries and associated outcome variables. In addition, we collected qualitative data to facilitate the interpretation of the findings from this quantitative data analysis and to identify strengths and limitations in design and implementation of GMIS.

### Data collection

Quantitative data were extracted from the national health information system on the monthly number of deliveries attended by trained health personnel or skilled attendants (in health centres, referral hospitals and at home) and the monthly number of home deliveries attended by traditional birth attendants, nationally and by health district, between January 2006 and December 2011. These data are routinely collected by individual health facilities and collated at the district level in a specific software package on a monthly basis. These reports are then sent to the provincial health office, which in turn forwards them to the central Ministry of Health. The expected number of births is estimated based on the population figure counted by the national census in 2008 with an annual growth rate of 1.54 % and a crude birth rate of 2.56 %.

In addition to the routine quantitative data, we also collected qualitative data in six selected health districts. These districts were selected based on the availability of major health financing schemes such as contracting, health equity funds and vouchers with the aim of covering all aspects or groups of districts (with none, one or several of these schemes). In each selected district, we interviewed the district supervisor for maternal and child health services and the chief of the technical bureau. These individuals are considered to be the most informed about GMIS and maternal health-related matters in the district. With help from the supervisor, we selected two health centres—one with relatively good delivery performance and another one with relatively poor performance. In each selected health centre, we interviewed midwives, the health centre chief and one of the community representatives. In addition, we conducted one focus group discussion with randomly selected women who had given birth (once or more) between 2006 and 2010 in each health centre catchment area. Based on the health centre’s coverage map, we first selected three villages according to geographical distribution: one closest to the health centre, one furthest away from the health centre and one in between. According to the village’s population size, in each village 9–12 eligible women (who had given birth between 2006 and 2010) were randomly selected and invited to participate in a focus group discussion. Moreover, in order to gain insights on GMIS policy, its potential effects and issues related to its design, implementation and monitoring, we conducted in-depth interviews with policy makers and managers from the Ministry of Health, development partners and non-governmental organisations in the capital city of Phnom Penh. They were purposively selected based on authors’ prior knowledge and through a snowball technique. We did not fix the number of key informants in Phnom Penh, but continued the interviews until we got an impression of saturation of messages.

Table 2 summarizes the sampling and number of respondents by location, type and method for qualitative data collection. In total, we conducted 12 focus group discussions with 124 women and interviewed 56 key informants, including 11 in Phnom Penh. The interviews with key informants in Phnom Penh were carried out by the first author (PI) and third author (KC) of this paper in both English and local languages, whereas the focus group discussions and interviews at the district level were conducted by two trained surveyors under close supervision by PI. We had guiding questions for key informant interviews and guidelines for focus group discussions.

**Table 2:** Sampling and number of respondents by location, type and method for qualitative data collection

Location	Health financing interventions	Number of respondents by type and method	
		In-depth interviews	Focus group discussions
OD1	Contracting; health equity fund; vouchers and community-based health insurance	1 OD MCH supervisor	2 focus group discussions with 24 women
		1 OD chief of the technical bureau	
		2 health centre chiefs	
		2 health centre midwives	
		2 community representatives	
OD2	Contracting; health equity fund; vouchers	1 OD MCH supervisor	2 focus group discussions with 19 women
		1 OD chief of the technical bureau	
		2 health centre chiefs	
		2 health centre midwives	
		2 community representatives	
OD3	Contracting; health equity fund	1 OD MCH supervisor	2 focus group discussions with 18 women
		2 health centre chiefs	
		2 health centre midwives	
		2 community representatives	
OD4	Health equity fund	1 OD MCH supervisor	2 focus group discussions with 24 women
		2 health centre chiefs	
		2 health centre midwives	
		2 community representatives	
OD5	Community-based health insurance	1 OD MCH supervisor	2 focus group discussions with 18 women
		1 OD chief of the technical bureau	
		2 health centre chiefs	
		2 health centre midwives	
		2 community representatives	
OD6	None	1 MCH supervisor	2 focus group discussions with 21 women
		2 health centre chiefs	
		2 health centre midwives	
		2 community representatives	
Phnom Penh	Not applicable	11 policy makers and managers: 6 from the Ministry of Health and 5 from development partners and NGOs	
TOTAL		56 key informants	12 focus group discussions with 124 women

*MCH* means maternal and child health*, OD* means operational district

### Data analysis

We first assessed the trends and changes in the proportion of deliveries attended by trained health personnel by location: in health centres, in referral hospitals and at home. Then we conducted an econometric analysis in order to assess the impact of GMIS. The absence of a baseline and the one-time nationwide launching of GMIS constrained us in terms of research design for the measurement of the effectiveness of the GMIS. Therefore, we used *segmented linear regressions,* as recommended, for example, by Lagarde [38] to assess the impact of GMIS. Authors of a recent Cochrane Review on performance-based financing argue that this method, although less robust, is still an acceptable method to assess the impact of a policy change with routine longitudinal data [15]. Our monthly data allowed us to assess the effect of the GMIS introduction on the following outcomes: monthly number of deliveries in public health facilities (facility deliveries) in all health districts and by group of districts with and without major health financing schemes; monthly volume of deliveries attended by trained health personnel (including home deliveries). The specification of the linear regression to be analysed was:

1 $$ {Y}_t={\beta}_0+{\beta}_1.t+{\beta}_2.{\mathrm{intervention}}_t+{\beta}_3. postslop{e}_t+{\varepsilon}_t $$

*Y*_*t*_ is the outcome variable at time *t*. Time is a continuous variable indicating time from the start of the study up to the end of the period of observation; intervention is coded 0 for pre-intervention time points and 1 for post-intervention time points (after October 2007); and postslope is coded 0 up to the last point before the intervention phase and coded sequentially from 1 thereafter. In this model, *β*_*0*_ captures the baseline level of the outcome at time 0 (beginning of the period); *β*_*1*_ estimates the structural trend or growth rate in utilisation, independently from the intervention; *β*_*2*_ estimates the immediate impact of the intervention on the outcome of interest (or the change in the level in the outcome of interest after the intervention); and *β*_*3*_ reflects the change in trend, or growth rate in outcome, after the intervention. We controlled for auto-correlation in the data series, by first performing a Durbin–Watson (DW) test to test the presence of first-order auto-correlation. The presence of first auto-correlation violates the ordinary least squares (OLS) assumption that the error terms are uncorrelated, meaning that the standard-errors and p-values are biased with the OLS estimator. The DW test statistic is as follows:

2 $$ DW=\frac{{\displaystyle {\sum}_{t=2}^T{\left({\varepsilon}_t-{\varepsilon}_{t-1}\right)}^2}}{{\displaystyle {\sum}_{t=1}^T{\varepsilon}_t^2}} $$

where T is the number of observations. The value of *DW* always lies between 0 and 4; *DW* = 2 indicates no autocorrelation. Small values of *DW* indicate that successive error terms are, on average, close in value to one another, or positively correlated.

Since auto-correlation was detected for all our four outcomes (*DW* < 1), the Prais–Winsten generalized least squares estimator [39] was used to estimate the regression. STATA 12 was used to perform all the estimates.

The qualitative data were manually coded and key messages were grouped by theme and by research question and analysed by group of districts. Findings from the analysis of qualitative data were carefully interpreted and triangulated with quantitative data analysis.

### Ethical considerations

This study received ethical approval from the National Ethics Committee for Health Research in Cambodia on 02 April 2012 with reference number 040 NECHR. The interviews were carried out by trained and professional surveyors. Prior to the interview, verbal consent was obtained from the interviewee. All the personal information of the interviewees has been kept confidential and no name has been used for the report or published papers.

## Results

### Results from descriptive analysis

Table 3 provides an overview of the proportion of annually reported deliveries by type of attendants (trained health personnel and traditional birth attendants) and by location (in health centres, in hospitals and at home) between 2006 and 2011. It shows that deliveries in public health facilities (facility deliveries) increased sharply from 18.9 % of the estimated number of birth in 2006 to 56.7 % in 2011. The increase in the proportion of deliveries in health centres (from 11.3 to 42.4 %) was much more substantial than in hospitals (from 7.6 to 14.3 %), while attended deliveries at home decreased from 21.1 to 14.8 % within the same period. This amounted to an overall increase in the proportion of facility deliveries by 200.1 % (275.4 % in health centres and 88.4 % in hospitals) and a decline in proportion of attended home deliveries by 30.1 % over this six-year period. Consequently, the proportion of deliveries attended by trained health personnel in all locations also increased by 78.6 % in the same period (from 40 % in 2006 to 71.5 % in 2011). The proportion of deliveries attended by traditional birth attendants declined dramatically from 29.3 % in 2006 to 5.4 % in 2011, resulting in a total decrease of 81.5 % between 2006 and 2011.

**Table 3:** Proportion of deliveries by type of attendants and location between 2006 and 2011

		By trained health personnel					By traditional birth attendants at home
	Year	In health centres	In hospitals	In public facilities	At home	All	
		(1)	(2)	(1) + (2)	(3)	(1) + (2) + (3)	
Deliveries as % of expected births in	2006	11.3	7.6	18.9	21.1	40.0	29.3
	2007	16.4	8.1	24.5	21.6	46.1	26.7
	2008	26.4	8.9	35.3	18.3	53.1	20.7
	2009	35.6	10.5	46.1	18.1	64.2	14.9
	2010	41.8	12.7	54.5	14.8	69.3	9.5
	2011	42.4	14.3	56.7	14.8	71.5	5.4
% of change between	2006–2007	44.8	6.9	29.6	2.4	15.2	−9.0
	2007–2008	59.2	8.8	42.5	−15.6	15.2	−22.3
	2008–2009	36.7	18.9	32.2	−0.8	20.9	−27.9
	2009–2010	17.3	20.9	18.1	−18.1	7.9	−36.7
	2010–2011	1.5	12.7	4.1	−0.4	3.2	−42.6
	2006–2011	275.4	88.4	200.1	−30.1	78.6	−81.5

The magnitude of annual change in proportion of facility deliveries jumped from 29.6 % between 2006 and 2007 to 42.5 % between 2007 and 2008, and then gradually slowed until 4.1 % between 2010 and 2011. In health centres, we found a similar pattern –a jump from 44 to 59.2 % and then a gradual decline until 1.5 %. Unlike in health centres, the magnitude of annual change in hospital deliveries progressively increased from 6.9 % between 2006 and 2007 to 20.9 % between 2009 and 2010 and then sharply declined to 12.7 % between 2010 and 2011. The magnitude of annual change in proportion of deliveries at home was variable.

### Results from segmented linear regressions

All models were run using an OLS estimator. The Durbin-Watson test statistic showed that there was first order auto-correlation (D-W test <1), meaning that the observations were positively correlated from one month to another; this violates the non-autocorrelation of residuals assumption, which leads to biased standard errors with the OLS estimator. In order to address this issue, we then performed the models using the Prais-Winsten generalized least squares estimator [39], which corrected the autocorrelation issue (D-W tests are then around 2). Overall, the use of this estimator slightly reduced the level and significance of the coefficients compared to the OLS one. The results described below and summarised in Table 4 all refer to the Prais-Winsten estimates.

**Table 4:** Impact of results-based financing on location and assistance of deliveries: Results from the segmented linear regression models

Dependent variable	Facility deliveries in districts with no other major financing scheme	Facility deliveries in districts with one or more other major financing scheme	Facility deliveries in all districts	All deliveries by trained health personnel in all districts
Number of health districts	19	58	77	77
Model	(1)	(2)	(3)	(4)
Constant	610.509***	3,710.119***	4,315.636***	10,366.858***
	(413.080–807.938)	(3,132.182–4,288.055)	(3,515.163–5,116.110)	(9,364.438–11,369.278)
Time(month)	36.235***	93.407***	132.135***	125.913***
	(20.380–52.090)	(47.549–139.264)	(70.328–193.942)	(53.338–198.488)
GMIS Intervention	489.685***	912.637***	1,330.918***	1,260.934**
	(219.206–760.163)	(306.590–1,518.685)	(488.316–2,173.519)	(181.964–2,339.903)
GMIS postslope	−1.307	83.973**	80.773*	54.600
	(−19.326–16.712)	(16.035–151.911)	(−5.097–166.644)	(−45.848–155.049)
Observations	72	72	72	72
R-squared	0.792	0.732	0.740	0.624
Durbin Watson original	0.985	0.688	0.711	0.792
Durbin Watson transformed	2.043	1.898	1.909	1.940

all regressions are using a Prais–Winsten estimator that corrects for data auto-correlation; *** p < 0.01, ** p < 0.05, * p < 0.1; confidence intervals (CI) in parentheses. Time variable is a sequence starting at 1 for the first month of the dataset (January 2006) to 72 for the last month (Dec 2011), its coefficient provides the secular trend of deliveries. GMIS Intervention and GMIS postslope are the level and trend variables for an intervention starting in October 2007: their coefficients represent respectively the change in level and the change in trend of deliveries after the introduction of GMIS. Other major health financing schemes include contracting and other performance-based financing, health equity funds, vouchers and community-based health insurance. R-Squared gives information about the goodness of fit of the model, the closer to 1, the better the data fit the model. Durbin-Watson (DW) statistic tests the presence of first-order auto-correlation. The presence of first auto-correlation violates the ordinary least squares (OLS) assumption that the error terms are uncorrelated, meaning that the standard-errors and p-values are biased with the OLS estimator. DW ‘original’ tests the presence of first-order auto-correlation with the OLS estimator, while DW ‘transformed’ tests it with the Prais-Winsten estimator. A value around 2 indicates no sign of auto-correlation. P-values and CI are based on a standard variance estimator.

**Fig. 1:**
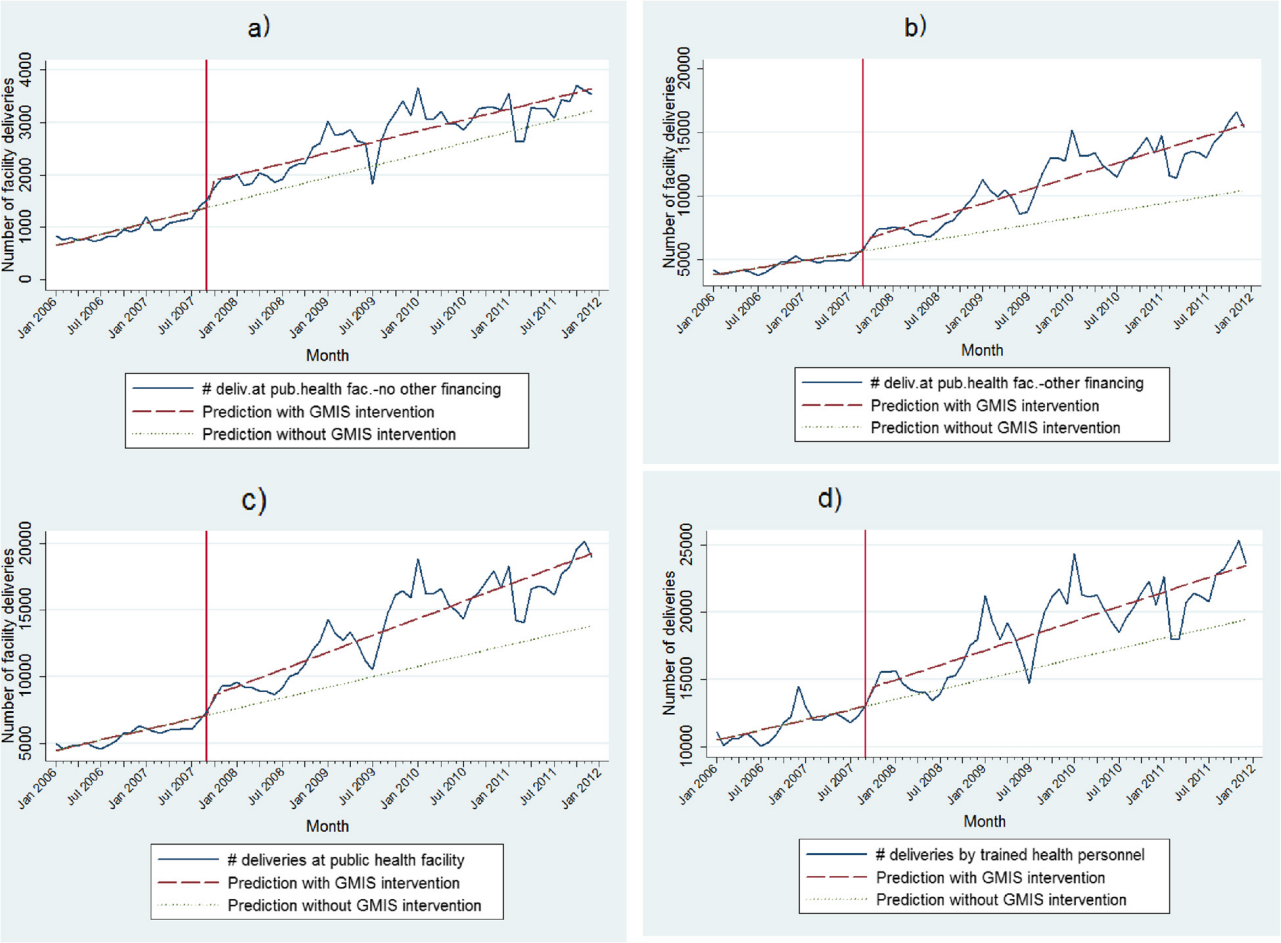
Impact of results-based financing on location and assistance of deliveries: Observed and predicted values. **a**) Facility deliveries in districts with no more other major financing intervention than GMIS. **b**) Facility deliveries in districts with one or more other major financing intervention than GMIS. **c**) Facility deliveries in all districts. **d**) All deliveries by trained health personnel in all districts

Model (1) in column one was performed on the data of 19 health districts which had no other major health financing scheme, such as contracting, health equity funds, vouchers, etc. The coefficient of the constant indicates that at the beginning of the period of observation, in this sub-region of Cambodia, there were on average 611 facility deliveries per month (*p*-value <0.01). There was a significant upward trend with an average of 36 more facility deliveries each month (*p*-value <0.01). Model (1) also shows that immediately after the intervention, the number of facility deliveries increased suddenly and significantly by 490 deliveries per month (*p*-value <0.01), but there was no significant change in the month-to-month trend (*p*-value >0.1). According to this data, the number of facility deliveries in October 2007 (the first month after the start of the intervention) was around 1,897, while it would have been 1,408 without the intervention, suggesting an increase in facility deliveries by 35 % the first month. Similarly, we estimate that the number of facility deliveries 12 months after the intervention was around 27 % higher than it would have been without the intervention. Figure 1a presents the raw data series of the outcome of interest, and the fitted results obtained from Model (1).

Model (2) was performed on the data of the other 58 health districts which had at least one other major health financing scheme. The coefficient of the constant indicates that at the beginning of the period of observation, there were on average 3,710 facility deliveries per month (*p*-value <0.01). There was a significant upward trend with an average of 93 more facility deliveries each month (*p*-value <0.01). Immediately after the intervention, the number of facility deliveries increased suddenly and significantly by 913 deliveries per month (*p*-value <0.01), followed by an upward trend of 84 deliveries each month (*p*-value <0.05). According to this data, the number of facility deliveries in October 2007 (the first month after the start of the intervention) was around 8,713, while it would have been 6,793 without the intervention, suggesting an increase of facility deliveries by 17 % the first month. Similarly, we estimate that the number of facility deliveries 12 months after the intervention was around 28 % higher than it would have been without the intervention. Figure 1b presents the raw data series of the outcome of interest, and the fitted results obtained from Model (2).

Model (3) provides estimates for the two groups of districts put together. Results indicate that at the beginning of the period of observation, there were on average 4,316 facility deliveries per month in Cambodia (*p*-value <0.01). There was a significant upward trend with an average of 132 more facility deliveries each month (*p*-value <0.01). Immediately after the intervention, the number of facility deliveries increased suddenly and significantly by about 1,331 deliveries per month (*p*-value <0.01). According to this data, the number of facility deliveries in October 2007 (the first month after the start of the intervention) was around 8,554, while it would have been 7,223 without the intervention, suggesting an increase of facility deliveries by more than 18.4 %. Similarly, we estimate that the number of facility deliveries 12 months after the intervention was around 15.4 % higher than it would have been without the intervention. Figure 1c presents the raw data series of the outcome of interest, and the fitted results obtained from Model (3).

Model (4) allows us to also consider deliveries attended by trained health personnel at home. Results indicate that at the beginning of the period of observation, there were on average 10,367 deliveries by trained health personnel per month in Cambodia (*p*-value <0.01), with a significant upward trend of 126 more deliveries each month (*p*-value <0.01). Immediately after the intervention, the number of deliveries by trained health personnel increased suddenly and significantly by 1,261 deliveries—a jump of 9.6 and 8.7 % at the first and 12^th^ month respectively after the introduction of GMIS. Figure 1d presents the raw data series of the outcome of interest, and the fitted results (dash line) obtained from Model (4).

We also ran segmented linear regressions on other outcome variables, including number of referrals of complicated deliveries from health centres to hospitals and results are available on request. Robustness checks were performed in order to check the reliability of these results. The linear relationship assumption between our outcomes and time was checked by adding a time-squared variable: it appeared to be not significant, confirming the linearity hypothesis. A model without trend effect (i.e. without the postslope variable as an explaining variable) ensures that there is a positive shift on the level of facility deliveries at the time GMIS was implemented. Placebo models, the models in which the intervention date was set at fake dates (one year before, two years after), confirm that the changes occurred concomitantly with the GMIS introduction. Models controlling for seasonal effects were also performed.

### Results from qualitative data analysis

#### Results from key informant interviews

The qualitative research corroborates the findings of the quantitative analyses. All the key informants stated that GMIS is a good government health policy and investment. They noted a dramatic increase in facility deliveries after the introduction of GMIS. A key informant, an expert in the field of maternal and child health, stressed that:

“*GMIS is a fantastic and very powerful policy. It has dramatically changed the delivery pattern in Cambodia. There has been a remarkable increase in deliveries in public health facilities, mainly in health centres. Giving birth in public health facilities rose from 11 % in 2000 to 60 % in 2010. Now, many health centres have around 20–25 deliveries per month. Before the introduction of GMIS, there was almost no case of delivery in many health centres. I have never seen any other countries with such incredible change. If you look at the data by year, you will see so beautiful increase. There is probably no example like this in the world…”*

Their statements are consistent with the theory of change underlying the GMIS policy. Incentives increase the income of midwives and other health personnel, and consequently their motivation and commitment to increase facility deliveries. Thanks to the incentives, midwives have changed their behaviour and practice from promoting home deliveries to promoting deliveries in public health facilities. Health centre midwives reported that they now prefer attending deliveries at the health centre, as attending home deliveries is more time consuming than health centre deliveries and is sometimes risky. Moreover, home deliveries do not necessarily generate more income than what they can earn from health centre deliveries. One midwife said:

“*Since I received the incentives (from GMIS), I have stopped attending deliveries at home. When I am asked by the woman or her family to do so, I will ask her or the family to bring her to the health centre. For home deliveries, I often have to go to the woman’s home, which is much more time consuming than waiting for the woman at the health centre and sometimes unsecured for me to travel at night time to woman’s home. Moreover, at home, I have to bear the risk and liability, whereas at the health centre, it is the collective responsibility. I used to get around USD15-USD20 per home delivery (which is sometimes difficult to get paid), which is comparable with what I earn from attending a delivery at the health centre—about USD10 from the GMIS plus user fees…”*

According to key informants in the six health districts, especially the district maternal and child health supervisors and chiefs of the technical bureau, GMIS not only increased facility deliveries and changed midwife behaviour and practices, but also triggered other changes at district and facility level. District and provincial health management teams seized the opportunity of midwives receiving incentives for delivery to reinforce their supervisions over health facilities especially for delivery services in order to prevent eventual falsification of reported cases. During the supervision, the delivery monitoring grid and signature of the commune chief was strictly checked for each reported facility delivery. Some even asked their respective health facilities to make delivery services free of charge (yet, we did not find this practice in the six health districts we investigated).

At health facility level, more attention has been paid to the reorganisation and improvement of delivery services. In a large majority of health centres, especially those with other major health financing schemes, 24-hour and seven-day-a-week services for delivery were put in place. They also organised on-call services outside working hours and at the weekend by posting cellphone numbers of midwives at the health centre or distributing them to women directly or through community health workers. It is now common that health centre midwives share their cellphone numbers with pregnant women during antenatal care and ask the women to call them when labour starts. Many health centres reported to have hired midwife assistant(s) to ensure the continuity of services. It was also reported that some health centres provided an in-kind incentive such as “*Sarong*” (Cambodian commonly used skirt) to clients in order to attract more pregnant women to deliver at the health centres.

All health centres reported to have shared part of the income from GMIS (between 30–50 %) with other personnel and provided cash incentives to traditional birth attendants and community health workers for referrals of pregnant women for delivery in their facilities. The amount of incentives often varied from USD1.25 to USD2.50 per referred case. This amount was found similar to what traditional birth attendants would earn from attending a home delivery. It was reported that in one health district, the health authority collaborated with commune councils to forbid traditional birth attendants to attend deliveries at home. For any home delivery attended by traditional birth attendants, if discovered, the responsible traditional birth attendants would get a USD25 fine and the money is put into the account of the commune.

In addition to the increased facility deliveries, almost all health centres also reported an increase in family planning services, antenatal care and postnatal care visits. In some health centres, even general outpatient consultations were also found to have increased considerably after the introduction of GMIS. This was mainly thanks to the increased availability and the commitment of midwives to their work and the overall improvement in the health centre.

Many key informants emphasized that the positive effects, especially the increased facility deliveries, were not entirely attributable to GMIS. Other factors including efforts in improving health infrastructure, equipment and supplies necessary for delivery services, and in training and capacity building of midwives and their deployment, also contributed to the improved health facility performance in general, and to increased facility deliveries. A national expert informed us that nearly one third of the health centres in Cambodia had no trained midwife; this number gradually decreased until the end of 2009 when all the health centres in the country had at least one trained midwife. At the same time, the number of basic and comprehensive emergency obstetric care facilities increased significantly. Moreover, many key informants expressed their view that the expansion of coverage of major health financing schemes such as contracting, health equity funds and vouchers was an important factor contributing to improving staff and facility performance, and hence, increased facility deliveries.

Along with the positive effects, some negative effects and limitations of GMIS were raised by key informants. Some shared anecdotal evidence and their concerns that in some health districts, especially those with no other major health financing schemes, there was no proper supervision and monitoring. In those districts, health facilities might over-report the number of facility deliveries or report home deliveries as facility deliveries. They might also pay commune chiefs to obtain their signatures. Possible delay in referrals of complicated cases from health centres to referral hospitals was also raised by some key informants in Phnom Penh, as the health centre would lose the incentive if they referred the pregnant woman to hospital. But this concern was rejected by almost all key informants at district and health centre level. They argued that the incentives of intentionally delaying referrals of complicated cases are not comparable to the risk of killing the woman, which is inhuman and intolerable. It was reported that in some health districts, some non-governmental organisations provide incentives to health centres for referrals of complicated cases to hospitals and introduced a number of measures for ensuring appropriate and timely referrals, including strengthening the monitoring system and ambulance services. Last but not least, almost all midwives and health centre chiefs interviewed complained about delay and incompleteness in disbursements of the incentives. The delay varies from two months to two quarters, and the cuts of the incentives at district and provincial level, for a number of reasons which could not be explained, were ranging from 10 to 20 % of the total revenues, depending on the context.

#### Results from focus group discussions with women

The increased facility deliveries and improved health facility performance were further confirmed by the women who participated in focus group discussions. They reported that almost all pregnant women in their villages, especially those in the districts with other major health financing schemes, are now giving birth at health centres. Some women with complicated pregnancy, those living closer to referral hospitals and those living far from hospitals but are willing and able to pay a higher cost go to hospitals for delivery. Some women declared that there is no more home delivery in their villages. This situation had gradually changed over a number of years and for a number of reasons: (i) public health facilities, mainly health centres in their areas, are in general performing better than they were some years ago; they are better equipped and cleaner; midwives are friendlier and more present, and if not present, can be easily called when needed; (ii) in some villages there are no traditional birth attendants, and if there are, they do not want to attend deliveries anymore, as they have been told not to attend delivery at home, but to refer pregnant women to health centres for which they will receive fair compensation; and (iii) women are increasingly aware of the benefits of giving birth in health centres and the risks of giving birth at home with traditional birth attendants, as a participant reported:

“*Giving birth in health centres has a lot of advantages. We believe that giving birth in a health centre is safer than doing so at home with traditional birth attendants, as midwives are better trained and more skilful than traditional birth attendants. In the health centre, there are more modern equipment and materials for delivery than at home. We can get our baby vaccinated after the delivery at the health centre; and it is easy to get a birth certificate for the baby. In case of difficult delivery, we can get help for referral to the hospital…*”

However, it was reported that for a number of reasons a small proportion of women still continue to deliver at home and sometimes with traditional birth attendants. One commonly reported reason was the fact that some pregnant women were not well prepared for delivery, and when it happened, it was too late to go to a health facility, and thus the babies spontaneously came out at home or on the way to the health facility. Other reasons were transportation, financial barriers and intra-household constraints. Women did not report any case of midwives asking for extra payments, but many of them reported to have paid extra as an act of gratitude or gratefulness to midwives for helping them deliver safely, as delivery is considered one of the most important and dangerous events in women’s lives, and therefore, traditionally named as *Chhlang Tonle* (crossing the river).

## Discussion

This study investigated four research questions through a mixed-methods design to primarily evaluate the impact of GMIS, a nationwide government implemented results-based financing scheme aiming at boosting facility deliveries in Cambodia.

The first question was whether the GMIS scheme did reach its objective. We had two constraints in carrying out the impact evaluation: (i) the availability of routine data only and (ii) the fact that the nationwide introduction of the scheme deprived us from any robust counterfactual. On the first point, we cannot exclude that one of the unintended effects of GMIS was to incentivise staff to over-report facility deliveries or at least to better report them. However, the similarity between the proportion of reported facility deliveries and deliveries attended by trained health personnel in 2010 in the routine data as indicated in Table 3 (55 and 69 % respectively) and those found by the population-based CDHS 2010 as shown in Table 1 (54 and 71 % respectively) seems to indicate that this problem has been marginal. Moreover, the quality of routine data, especially for maternal and child health indicators, has significantly improved since 2006 [40, 41]. On the second point, we cannot exclude that other concomitant phenomena also contributed to the sharp increase in the number of facility deliveries and deliveries by trained health personnel. Many key informants spontaneously mentioned that the rapid progress in Cambodia with regards to reducing maternal mortality is the outcome of a multifaceted strategy, as found by a recent study [42]. Still, the coefficients of the regressions are strongly significant. The interrupted time series analyses confirm the pre-existing view—shared by all our informants—that GMIS did significantly help with the overall country strategy to improve health system performance and boost facility deliveries, and consequently, deliveries attended by trained health personnel.

Although the interrupted time series is considered an acceptable method to assess the impact of a policy change with routine longitudinal data, this approach has an obvious limitation. While it can confirm a shift in the outcome variable, at and after the implementation of the intervention, and can even give its magnitude, it does not guarantee that the intervention was the causal determinant of that shift. Concomitant reforms or events might also have had an influence on the assessed outcome variable. Our complementary qualitative insights helped us to interpret the quantitative results and thus minimized limitations of the latter.

As a result of our qualitative analysis, we also have a better view on what happened in the ‘black box’. The narrative report by key informants was that incentives increased the income of midwives and other health personnel, and consequently their motivation and commitment to deliveries in public health facilities. Thanks to the incentives, midwives have changed their behaviour and practice from promoting home deliveries to promoting facility deliveries. This positive change after the introduction of GMIS was further evidenced by 124 women who had given birth (once or more) since 2006 and participated in the focus group discussions. They reported that now most pregnant women in their villages go to health centres for deliveries. Key informants also gave us some insights into other factors which may have contributed to the increased facility deliveries: they mentioned improvement of health infrastructure, equipment and supplies necessary for delivery services, efforts in training and capacity building of midwives and their deployment, as evidenced by the considerable decline in number of health centres with no trained midwife from 223 in 2006 to zero by 2009. In addition, the expansion of coverage of major health financing interventions such as contracting, health equity funds and vouchers was also considered an important factor contributing to improving staff and facility performance, as pointed out by Liljestrand and Sambath [7]. A recent study confirmed the impact of reproductive vouchers on facility deliveries in Cambodia [31].

A second question was concerned with the possible spill-over effects of GMIS. Our research, the qualitative work in particular, gives some insights. As demonstrated by the statements of key informants in the six selected health districts, the introduction of GMIS not only increased facility deliveries and deliveries by trained health personnel and influenced midwives’ behaviour and practices, but also triggered other changes at district and facility level. These included improved supervisions and monitoring from district and provincial teams over their respective health facilities, mainly on maternal and child health related activities; better organised health services with often 24-hour and seven-day-a-week services (or at least on-call) for delivery; better teamwork among staff who fairly share incentives and related tasks among them; and improved referrals of pregnant women from villages to health centres for delivery by providing education and incentives to traditional birth attendants and community health workers. Thanks to this overall improvement, almost all health centres reported an increase in family planning services, antenatal care and postnatal care visits. In some health centres, even general outpatient consultations were also found to have increased considerably after the introduction of GMIS. According to the results from segmented linear regressions, the introduction of GMIS appears to also have a positive effect on referrals of complicated deliveries from health centres to hospitals. Although many key informants considered key interventions and strategies highlighted in the ‘Fast Track Initiative’ as key factors contributing to improved staff and facility performance and increased deliveries in public health facilities; some saw these as being an indirect result of the introduction of GMIS. Many of these interventions and strategies, including the Fast Track Initiative itself, were developed or intensified after the introduction of GMIS to make this policy sufficiently successful to achieve MDG 5.

The third question related to the possibility that the effectiveness of GMIS varies across district health systems. The comparison between Models (1) and (2) indicates that GMIS had a stronger short term effect in districts with no other major financing scheme. As time goes by, however, this advantage seems to vanish, as districts with another major financing scheme have a steeper slope. This was further explained by results from key informant interviews. Changes in health service organisation (e.g. 24-h services) which is key to increased facility deliveries, tends to happen more in health districts with contracting whereas stronger monitoring and supervision were seen in districts with a third party purchaser for other contracting, health equity funds, vouchers and community-based health insurance than in those without such arrangements. According to women participating in the focus group discussions, in health districts with long-lasting and multiple external support, including those with other major health financing schemes, there are almost no women giving birth at home with traditional birth attendants. This was not the case in health districts without such interventions, especially for disadvantaged health centres (remote and poor leadership). This stronger effect of GMIS in health districts with other major health financing schemes strongly suggests their complementarity and synergy.

Our last question was related to the possible shortcomings or undesirable side-effects of the GMIS. According to the literature, an output-based or performance-based incentive scheme like GMIS can also have negative or undesirable effects, including distortions, gaming or fraud. Financial incentives may be stolen or misused or cause recipients to undermine or ignore unrewarded tasks. Some providers may only show changes in reporting (improving or falsifying figures) without necessarily changing practices. Furthermore, this approach can increase dependency on financial incentives, dilute professionals’ intrinsic motivation, lead to demoralization due to feelings of injustice, and can increase the administrative burden and costs due to bureaucratisation [10, 17, 43].

Some key informants in Phnom Penh shared anecdotal evidence and their concerns that in some health districts, especially in the districts with no third party purchaser, there was no proper supervision and monitoring. In these districts, health facilities might over-report the number of facility deliveries or report home deliveries as facility deliveries. They might also pay commune chiefs to obtain their signatures. However, our study did not find any evidence on this. As discussed above, the consistency of the routine data over the study period and its comparability with the CDHS 2010 data suggests that the over-reporting of delivery cases by health facilities, if any, is negligible.

Possible delay in referrals of complicated cases from health centres to referral hospitals was also raised by some key informants in Phnom Penh, as health centre midwives would not receive the incentive if they referred the woman to hospital. But this concern was rejected by almost all key informants at health district and health centre level. Only one maternal and child health supervisor said she observed a tendency to keep difficult cases at one health centre in her district, but after explanation and discussion with midwives there, things have now changed. Moreover, our regressions show a positive upward trend of monthly number of referrals of complicated deliveries after the introduction of GMIS. Positive results have been observed from the pilot experience in many health districts in Banteay Meanchey and Battanabang provinces where the introduction of incentives together with improved monitoring and an ambulance for referrals have led to an increase in appropriate and timely referrals of complicated deliveries to hospitals. These results have led some key informants from NGOs to strongly suggest that the government should consider paying incentives for such referrals if GMIS is to be continued. However, one can argue that such incentives alone will not address the problem of delay in referrals, if any, without other accompanying measures such as improved monitoring and ambulances. In the absence of effective monitoring, such incentives can also lead to over-referrals because it is theoretically easier for a midwife to refer a pregnant woman to a referral hospital and get the incentive rather than keeping the woman to deliver at the health centre. Nevertheless, technically it could be good to introduce the incentives and other necessary measures for ensuring appropriate referrals of complicated deliveries from health centres to referral hospitals if the government is willing and able to do so. The is because the reported referrals as percentage of total reported facility deliveries in Cambodia (the highest rate was about 7 % in 2011), remains low as compared to a general estimate that around 15 % of all pregnant women might develop a potentially life-threatening complication [44]. However, such an incentive system should be closely monitored to prevent unnecessary referrals.

Another common concern is that excessive increase in institutional deliveries can lead to an increase in unnecessary C-sections. We did not investigate this issue as it is beyond the scope of this study. However, the current C-section rate in Cambodia, despite an increasing trend, remains at around 4 % of the total number of reported institutional deliveries.

Some key informants even said that providing incentives to midwives for facility deliveries could undermine their attention to other services, especially family planning. To increase the number of deliveries, it is better to have fewer women using modern contraceptive methods. However, as discussed above, interviews with key informants at health district and health centre level rather found the opposite.

Last but not least, almost all midwives and health centre chiefs interviewed complained that late and incomplete disbursement of the incentives was common. The delay varied from two months to two quarters, and cuts at district and provincial levels ranged from 10 to 20 % of the total revenues, depending on the context. If GMIS is to be continued, further improvement in incentive disbursement is needed.

We believe that our findings, with their strengths and limitations, are quite consistent with the current state of knowledge on RBF in low-income countries. The most remarkable fact is that our study confirms that RBF seems to work very well with respect to institutional deliveries, a finding already observed in several African countries such as Rwanda [45] and Burundi [46]. This could indicate that in many low-income countries, the low institutionalisation of deliveries is not only the result of demand-side barriers (e.g. user fees, distance, limited education of women…), but also stemming from supply side barriers.

## Conclusions

Despite some weaknesses in the methods, our findings strongly suggest that GMIS is an effective mechanism to complement other interventions to improve health system performance and boost deliveries by trained health personnel in public health facilities, especially at health centres, thereby contributing to the reduction of maternal mortality. In addition to the findings on the positive impact of GMIS, this study also highlighted a number of strengths and limitations of this scheme, including the context and other factors that make it function effectively. These factors provide useful lessons for Cambodia to further improve GMIS and for other developing countries to implement similar output-based financing mechanisms.

The introduction of GMIS in late 2007 together with other efforts to remove supply and demand barriers to professional maternal health services, has led to considerable improvements in public health facilities and a steep increase in institutional and assisted deliveries. GMIS is no doubt a key factor contributing to this achievement, but the real extent of its contribution cannot be assessed. A part from the GMIS, other interventions such as the rapid expansion of midwife coverage to all health centres; improvement of continuum of care for mothers, newborns and children; improvement of referral system; development of delivery waiting rooms at health centres in rural areas; and the expansion of contracting, health equity funds and vouchers are also contributing to this major change.

Besides the positive effects, GMIS has several operational issues and limitations that need to be addressed. Improving the financial incentive disbursement and monitoring system is crucial for the effectiveness of this output-based financing scheme. Careful implementation of such a scheme, as part of a broader package of health care reforms aimed at improving access to skilled birth attendants and emergency obstetric care, as indicated in the Ministry of Health’s Fast Track Initiative, could help address the limitations of GMIS. It could also improve its effectiveness in complementing other interventions to reach the ultimate goal of reducing maternal mortality and thus achieving MDG 5. As emphasized by key informants, GMIS reflects the strong commitment of the Royal Government of Cambodia to MDG 5 and such commitment should be continued and maintained. The comprehensiveness of the safe motherhood strategy of Cambodia could also be a source of inspiration for other low-income countries.
